# Circadian Preference, Sleep Quality, and Health-impairing Lifestyles Among Undergraduates of Medical University

**DOI:** 10.7759/cureus.2856

**Published:** 2018-06-21

**Authors:** Anil Gangwar, Sunita Tiwari, Anita Rawat, Ajay Verma, Kalpana Singh, Surya Kant, Ravindra Kumar Garg, Prithvi K Singh

**Affiliations:** 1 Physiology, King George Medical University, Lucknow, IND; 2 Department of Physiology, King George Medical University Lucknow,INDIA; 3 Department of Physiology, Hind Institute of Medical Sciences,sitapur, India; 4 Pulmonary Critical Care Medicine, King George Medical University Lucknow,INDIA; 5 Department of Biochemistry, King George Medical University Lucknow,INDIA; 6 Department of Respiratory Medicine, King George Medical University Lucknow,INDIA; 7 Department of Neurology, King George Medical University, Lucknow, IND; 8 Center for Advance Research/Cytogenetic Unit, King George Medical University, Lucknow, IND

**Keywords:** chronotype, medical students, epworth sleepiness score, morningness-eveningness, sleep quality

## Abstract

Background/Aims

Health-impairing lifestyle during adolescence is an important marker for poor health. An unhealthy lifestyle may lead to physical and psychological problems in adulthood. Most of the prior researches were done among the adult population. Therefore, we assessed the chronotype pattern and correlation of health-impairing lifestyles with sleep quality according to circadian typology in medical students.

Material and Methods

In this cross-sectional research, a total of 203 subjects were enrolled. All subjects were divided into definite evening chronotype (n = 73), intermediate chronotype (n = 87), and definite morning chronotype (n = 43). Electronic media use at bedtime and duration of media use, the timing of dinner, smoking, tobacco chewing, and alcohol consumption were assessed with the help of a preformed proforma. Physical activity, sleep quality, daytime sleepiness, and chronotype were assessed by International Physical Activity Questionnaire (IPAQ), Pittsburgh Sleep Quality Index (PSQI), Epworth Sleepiness Score (ESS), and Morningness-Eveningness Questionnaire Self-assessment version (MEQ-SA), respectively.

Results

Subjects of the evening chronotype were suffering more with poor sleep quality. Evening chronotype had a significant (p < 0.05) positive correlation between poor sleep quality and sex, tobacco smoking, alcohol drinking, type of diet, and timing of dinner.

Conclusion

Circadian typology demonstrated the significant correlation of health-impairing lifestyles with sleep quality. From this observation, it might be a better way to plan their daily activities, in accordance with their chronotypes, benefiting not only their academic performance but also their quality of life.

## Introduction

The relationship between psychological and behavioral variables with different sleep-wake habits was studied in human beings [1]. Morningness-eveningness refers to diurnal preferences of an individual for activity and alertness in the morning and evening. Various biological, genetic and psychosocial components are associated with the morningness-eveningness [2]. Initially, the Morningness-Eveningness Questionnaire [3] was used to distinguish between the two extreme diurnal types: morning type and evening type [4], and since then this questionnaire has been modified a number of times [5]. Some research involved morning type and evening type [3], whereas in other research, morning, intermediate, and evening types were studied [6]. Some important factors such as age, sex [7-8], personality [9], food intake [10], sleeping habits, smoking [11] and physical activity [12] were associated with morningness-eveningness. Previous studies have found that in adults [13] and adolescents [14-15] both circadian typology were associated with tobacco smoking. It was found that individuals with eveningness chronotypes have higher tendency to consume psychoactive substances as compared morningness and intermediate chronotype [10]. Some studies have found that subjects with eveningness chronotype have a higher tendency of having health-impairing lifestyle compared to morningness chronotype [12, 15]. However, the majority of these studies involved adult subjects. Therefore, it would be important to focus on the adolescent population in order to understand the associations between chronotype and health impairing lifestyle in a younger population. Regardless of the individual chronotype, the sleep-wake cycle should be synchronized in every individual. In undergraduates of the medical university, sleep-wake cycle is modified by various factors, such as curricular load, extracurricular activities, physical and emotional stress and pressure for high academic performance [16]. Therefore, it is necessary to focus on this subset of adolescents. The aims of this study were (a) to identify chronotypes pattern in medical undergraduates empirically, (b) to test the correlation between sleep quality and health impairing lifestyle. Based on our literature review, our hypothesis was that undergraduates of a medical university with morningness chronotype would be associated with lower likelihood of smoking, alcohol use, and electronic media use.

This research work was presented at Int. Conf. on Sleep Medicine and Research (Goasleep2017), Dona Paula, India, Sept. 22-23, 2017 (Gangwar AK, Rawat A, Tiwari S, Garg R, Kant S: Correlation of Chronotype with Sleep Quality, Demographic and Life Style Factors in Asymptomatic Healthy Adolescents - poster 38; http://link.springer.com/content/pdf/10.1007/s41782-017-0013-x.pdf.)

## Materials and methods

In this cross-sectional study, a total of 203 subjects were enrolled among first-year medical undergraduates on the basis of inclusion and exclusion criteria. Subjects with any known sleep problem, oro-nasal disease, head injury, and not willing to participate in the study were excluded from the study. Subjects with any chronic illness, such as diabetes mellitus, hypertension, and chronic respiratory disease, were also excluded from the study. All subjects were divided into three groups: group 1 (definite evening chronotype, n=73), group 2 (intermediate chronotype, n = 87), and group 3 (definite morning chronotype, n = 43) on the basis of the Morningness-Eveningness score [3]. Informed written consent was taken from all subjects after ethical clearance by the ethical committee of King George's Medical University (KGMU), Lucknow, India. The participants were then invited to respond to questionnaires. Complete clinical evaluation of subjects was done on the basis of a preformed proforma. Anthropometric measurements, such as height, weight, body mass index (BMI), and neck circumference, were taken by trained nursing staff. Type of electronic media used, whether used at bedtime or not, and duration of electronic media use (hours per week) were assessed on a subjective basis. Type of food (vegetarian or non-vegetarian), the timing of dinner, tobacco smoking, tobacco chewing, and alcohol consumption were also assessed subjectively with the help of the preformed proforma. The level of physical activity, sleep quality, daytime sleepiness, and chronotype were assessed by International Physical Activity Questionnaire (IPAQ) [17], Pittsburgh Sleep Quality Index (PSQI) [18], Epworth Sleepiness Score (ESS) [19], and Morningness-Eveningness Questionnaire Self-assessment version (MEQ-SA) [3], respectively. The Statistical Package for Social Sciences (SPSS) (IBM SPSS Statistics, Armonk, NY), version 21 was used for data analysis. Data were summarized as the mean ± standard deviation (SD) and the crude odds ratios (OR) of the demographic and lifestyle factors of all study subjects were calculated for poor sleep quality. Karl Pearson's correlation coefficient between demographic and lifestyle factors with poor sleep quality was calculated for all the three groups.

## Results

Demographic and factors affecting the lifestyles of groups 1, 2, and 3 are summarized in Table 1. No statistically significant difference was observed in age, sex, weight, height, neck circumference, tobacco chewing, tobacco smoking, alcohol drinking, physical activity, and duration of electronic media use (hours per week) among the study groups, although a statistically significant difference was found in BMI (p < 0.029), sleep quality (p < 0.001), sleep duration (p < 0.001), electronic media use at bedtime (p < 0.004), and timing of dinner (p < 0.001) among study groups. On comparing the groups, we saw that subjects of group 1 were suffering more with poor sleep quality compared to subjects of groups 2 and 3.

**Table 1:** Demographic and Lifestyle Factors of Study Subjects Data are represented as mean ± standard deviation, n (%), and ratio. * significant (p < 0.05); cm: centimetre; hrs: hours; kg: kilograms; n: number; SD: standard deviation; wk: week

	Group 1 (n = 73)	Group 2 (n = 87)	Group 3 (n = 43)	p-Value
Age (years)	18.21 ± 0.67	18.90 ± 0.68	18.05 ± 0.65	0.395
Sex				
Male	47 (64.38%)	57 (65.52%)	27(62.79%)	0.954
Female	26 (35.62%)	30 (34.48%)	16 (37.21%)	
Weight (kg)	64.49 ± 8.53	63.62 ± 8.35	61.95 ± 8.30	0.293
Height (cm)	167.27 ± 8.17	168.02 ± 8.17	168.21 ± 8.57	0.793
BMI	23.06 ± 2.45	22.50 ± 2.15	21.89 ± 2.27	0.029^*^
Neck Circumference (cm)	37.41 ± 2.40	37.47 ± 2.36	37.30 ± 2.58	0.933
Sleep Quality				
Good	7 (9.6%)	19 (21.84%)	26 (60.46%)	< 0.001^*^
Poor	66 (90.4%)	68 (78.16%)	17 (39.54%)	
Sleep Duration (hrs)				
≥ 7 hrs	8 (10.99%)	10 (11.5%)	10 (23.25%)	<0.001^*^
6 - 7 hrs	15 (20.55%)	16 (18.4%)	25 (58.14%)	
< 6 hrs	50 (68.5%)	61 (70.1%)	8 (18.6%)	
Tobacco Smoking				
Yes	30 (41.1%)	30 (34.48%)	10 (23.25%)	0.148
No	43 (58.9%)	57 (65.52%)	33 (76.75%)	
Tobacco Chewing				
Yes	4 (5.48%)	5 (5.75%)	1 (2.33%)	0.672
No	69 (94.52%)	82 (94.25%)	42 (97.67%)	
Alcohol Drinking				
Yes	30 (41.1%)	33 (37.93%)	9 (20.93%)	0.074
No	43 (58.9%)	54 (62.07%)	34 (79.07%)	
Physical Activity				
Inactive	2 (2.74%)	1 (1.15%)	1 (2.32%)	0.904
Minimally active	61 (83.56%)	72 (82.76%)	34 (79.07%)	
HEPA active (Health enhancing physical activity)	10 (13.69%)	14 (16.09%)	8 (18.6%)	
Electronic Media Use (hrs/wk)	18.87±5.85	20.45±5.99	18.85±5.98	0.174
Electronic Media Used (At bedtime)				
Yes	57 (78.1%)	71 (81.61%)	16 (37.21%)	< 0.001^*^
No	16 (21.9%)	16 (18.39%)	27 (62.79%)	
Timing of Dinner				
< 9 PM	18 (24.66%)	51 (58.62%)	28 (65.12%)	< 0.001^*^
≥ 9 PM	55 (75.34%)	36 (41.38%)	15 (34.88%)	

Crude OR of demographic and lifestyle factors for poor sleep quality are shown in Table 2. All the subjects with an age > 20 years showed high OR compared to subjects with an age ≤ 20 years for poor sleep quality. Short sleep duration (< 6 hours) showed a high OR of 1.24 (0.75 - 2.04), while long sleep duration (≥ 7 hours) showed a lower OR of 0.94 (0.45 - 1.97) for poor sleep quality. The long duration of electronic media use (> 14 hours/ week) and electronic media use at bedtime showed higher OR compared to less duration of electronic media use (≤ 7 hours/ week) and electronic media not used at bedtime, respectively. Late night dinner (≥ 9 pm) was strongly associated with poor sleep quality compared to dinner taken before 9 pm. A high ESS was strongly associated with poor sleep quality compared to a low ESS.

**Table 2:** Crude Odds Ratios (OR) of Demographic and Lifestyle Factors for Poor Sleep Quality CI: confidence interval; cm: centimetre; EM: electronic media; ESS: Epworth Sleepiness Score; HEPA: health-enhancing physical activity; hrs: hours; P: probability

	Poor Sleep Quality Frequency (%)	OR (CI)	p-Value
Age			
≤ 20 years	40.39	1	
> 20 years	32.51	1.03 (0.67 - 1.58)	0.975
Neck Circumference (cm)			
Male			
< 37	12.81	1	
≥ 37	33.00	1.09 (0.61 - 1.97)	0.844
Female			
< 34	4.93	1	
≥ 34	22.17	0.90 (0.36 - 2.27)	0.823
Physical Activity			
Inactive	1.48	1	
Minimally Active	59.61	0.97 (0.21 - 4.40)	0.964
HEPA	11.82	1.00 (0.20 - 4.90)	1
Sleep Duration (hrs)			
≥ 7	8.37	0.94 (0.45 - 1.97)	0.879
6 - 7	17.73	1	
<6	46.80	1.24 (0.75 - 2.04)	0.467
EM used (hrs/week)			
≤ 7	2.46	1	
8 - 14	8.37	1.33 (0.32 - 4.07)	0.847
> 14	62.07	1.79 (0.38 - 3.69)	0.777
EM Used at Bedtime			
Not Used	14.78	1	
Used	58.13	1.32 (0.79 - 2.20)	0.349
Timing of Dinner			
< 9 PM	31.03	1	
≥ 9 PM	41.87	1.24 (0.81 - 1.89)	0.389
ESS			
0 - 9	50.74	1	
10 - 11	9.36	1.53 (0.78 - 3.04)	0.29
12 - 24	12.81	1.53 (0.84 - 2.79)	0.209

Correlation between demographic and lifestyle factors with poor sleep quality in groups 1, 2, and 3 are shown in Table 3. There was a significant positive correlation between poor sleep quality and sex, tobacco smoking, alcohol drinking, diet (vegetarian or non-vegetarian), and timing of dinner. There was a significant negative correlation between poor sleep quality and age and electronic media use duration (hours/ week). Weight, height, BMI, and neck circumference have a negative correlation with poor sleep quality but statistically were not significant in the subjects of group 1. There was a significant positive correlation between poor sleep quality and age, sex, tobacco smoking, alcohol drinking, diet, electronic media use duration, and ESS. Other demographic and lifestyle factors were positively correlated with poor sleep quality but statistically were not significant in the subjects of group 2. There was a significant positive correlation between poor sleep quality and age, sex, and tobacco smoking but no association was present between poor sleep quality and tobacco chewing, alcohol drinking, and electronic media use at bedtime. Weight, height, the timing of dinner, neck circumference, sleep duration, and electronic media use duration were negatively correlated with poor sleep quality but the correlation was not statistically significant in the subjects of group 3. 

**Table 3:** Correlation Between Demographic and Lifestyle Factors with Poor Sleep Quality in Groups 1, 2, and 3 * = Significant (p < 0.05) BMI: body mass index; EM: electronic media, ESS: Epworth sleepiness score

Demographic and lifestyle variable	Group 1 (Karl-Pearson’s correlation coefficient)	Group 2 (Karl-Pearson’s correlation coefficient)	Group 3 (Karl-Pearson’s correlation coefficient)
Age	-0.333*	0.445*	0.739*
Sex	0.885*	0.785*	0.511*
Weight	-0.163	0.113	-0.112
Height	-0.155	0.028	-0.159
BMI	-0.040	0.099	0.024
Smoking	0.508*	0.446*	0.511*
Tobacco	0.214	0.136	0
Alcohol	0.427*	0.447*	0
Diet	0.535*	0.374*	0.366
Timing of Dinner	0.261*	0.033	-0.198
Neck Circumference	-0.244	0.067	-0.391
Physical Activity	0.071	0.079	0.276
Sleep Duration	0.149	0.178	-0.130
EM used (hrs/wk)	-0.361*	0.377*	-0.061
EM at bedtime	0.205	0.111	0
ESS	0.232	0.262*	0.203

## Discussion

Our results showed that the majority of study subjects could be classified as intermediate type, followed by evening type and the least frequent one was the morning type. The results suggested that there was a substantial relationship between the chronotypes and the quality of sleep in the undergraduate students under study. The evening-type individuals showed worse quality of sleep when compared with the morning and intermediate types of individuals. Similar results were also found in other studies [20-21]. In this study, we found a high prevalence of poor quality of sleep among undergraduate students, corroborating previous researchers who also found the high prevalence of poor sleep quality among medical students. There are several pieces of evidence that medical students are more prone to sleep-wake disturbances [16]. This susceptibility to sleep disorders might be explained by the presence of a curricular load and school schedule disrespecting the morningness-eveningness balance among medical students [16]. Age and gender were significantly correlated with the poor sleep quality in the subjects of groups 1, 2, and 3. A study from Japan confirmed that girls had a longer sleep initiation time compared to boys [22]. Gender influences were present in sleep habits and sleep characteristics, but the results were inconsistent among studies [22-23]. Our results demonstrated that there are higher chances of smoking and alcohol use in evening type individual as compared to morning type individual. Similar findings were reported by previous studies [13, 15, 24]. An association between chronotypes and health-impairing lifestyles can be explained by different pathways. The first hypothetical pathway is innovation seeking [9]. Another pathway is through the association between chronotypes and markers of endogenous circadian rhythmicity [1, 14]. The misalignment of social and biological time might cause stress, which possibly leads to adaptive and maladaptive coping, such as tobacco smoking and alcohol drinking [13]. In the evening chronotype (electronic media use) and in the intermediate chronotype (duration of electronic media use) were significantly correlated with poor sleep quality. Use of social media sites was the most important factor that affected sleep quality. Studies have suggested that sleep duration and sleep quality were associated with social media and electronic media use [25-26]. It may be due to reduced total sleep time [27] or due to bright light emitted by the electronic media that may delay circadian rhythms [28]. In the present study, we also found that poor sleep quality was positively and significantly associated with the type of diet (vegetarian/non-vegetarian) in the evening and intermediate chronotypes. Reduced total sleep time and poor sleep quality were associated with a low intake of vegetables [29]. The present research highlighted that evening-type adolescents have a higher risk of health-impairing lifestyles and also have an unhealthy eating pattern, characterized by a late night dinner. Replications of these findings are required in future research to explain the associations between circadian typology and health-impairing lifestyles. Future research should identify possible interventions according to the circadian typology to promote a healthy lifestyle and improve their quality of life. Our findings have public health importance for the large number of adolescents who have social jetlag and are sleep-deprived. We strongly advocate to promote the awareness in the medical field, as well as in the society, for the importance of circadian typology and the negative consequences of challenging our internal clock.

The present study was not without limitations. First, the present sample was limited to urban adolescents, and therefore, its generalization to a rural population is restricted. Second, this study was based only on self-report measures, which might be prone to memory and response biases.

## Conclusions

The chronotypes demonstrated a significant association with the quality of sleep. Evening-type individuals showed a poor quality of sleep in comparison to the morning individuals, as well as the intermediate individuals. There was a high prevalence of poor quality of sleep and excessive diurnal sleepiness among the medical students. Furthermore, chronotypes also demonstrated the significant correlation of health-impairing lifestyles, such as tobacco smoking, alcohol drinking, and late night dinner, with sleep quality. From the observation of the impact of circadian biology among the medical students, there might be a better way to plan their daily activities, according to their chronotypes, benefiting not only their academic performance but also their quality of life.
